# Intercalated duct cell is starting point in development of pancreatic ductal carcinoma?

**DOI:** 10.1186/1477-3163-4-9

**Published:** 2005-07-14

**Authors:** Ryo Wada, Kaoru Ogawa, Toshikazu Yamaguchi, Takayuki Tanizaki, Michio Matsumoto

**Affiliations:** 1 Department of Pathology, Juntendo University Shizuoka Hospital, Shizuoka, Japan; 2 Department of Pathology (I), Juntendo University, School of Medicine, Tokyo, Japan; 3 Department of Internal Medicine, Juntendo University Shizuoka Hospital, Shizuoka, Japan; 4 R & D Center, Biomedical Laboratories, Inc., Kawagoe, Saitama, Japan

**Keywords:** pancreatic cancer, intercalated duct, mucous gland hyperplasia, PanIN, K-ras, histogenesis, molecular analysis

## Abstract

**Background:**

Although it is well known that the pancreatic ductal carcinoma may develop having a relationship to the mucous gland hyperplasia (MGH) with atypia (PanIN-1B by PanIN system), the starting point of this atypical MGH is unclear. To know it, we examined the pancreas tissue using many methods described below.

**Methods:**

1. Twenty-seven surgically resected pancreas tissue specimens, including pancreatic ductal carcinomas (PDC), chronic pancreatitis and normal pancreas, were investigated using immunohistochemical stainings for MUC1, MUC6, 45M1, Ki67 and p53. 2. DNA extraction and analysis of K-ras mutation at codon 12 using microdissection method: The paraffin blocks with 16 regions including the intercalated duct cell (IC) adjacant to the atypical MGH were prepared for DNA extraction. Mutation of K-ras codon 12 was analized and compared in enriched polymerase chain reaction-enzyme-linked minisequence assay (PCR-ELMA).

**Results:**

1. In the normal pancreas, although no positive cell was seen in 45M1, p53, Ki67, the cytoplasm of IC were always positive for MUC1 and sometimes positive for MUC6. In the pancreas with fibrosis or inflammation, MGH was positive for MUC6 and 45M1. And atypical MGH was positive for MUC1, MUC6 and 45M1. Some IC adjacent to the atypical MGH was positive for Ki67 as well as atypical MGH. The carcinoma cells in all cases of PDC were diffusely positive for MUC1, 45M1, p53 and Ki67, and focally positive for MUC6. 2. In K-ras mutation, we examined the regions including IC adjacent to the atypical MGH, because the immunohistochemical apomucin stainings of these regions resembled those of PDC as decribed above. And K-ras mutation was confirmed in 12 of 16 regions (75%). All mutations were a single mutation, in 6 regions GTT was detected, in 4 regions GAT was detected and in 2 region AGT was detected.

**Conclusion:**

Some intercalated duct cell may be the starting point of the pancreatic ductal carcinoma, because the exhibitions of mucin expressions, Ki67, p53 and K-ras mutation in some intercalated duct cell resembled those of mucous gland hyperplasia or pancreatic ductal carcinoma.

## Background

The pancreatic ductal carcinoma (PDC) is fatal, even if its size is very small [1,2]. Therefore, it is very important to know the characteristics of pre-cancerous lesion of PDC for the preventive medicine and the early detection of PDC.

It has been well known that the mucous gland hyperplasia (MGH) (goblet cell metaplasia) is one of pre-cancerous lesion of PDC [3-5], and today, the histogenesis of PDC has been accepted by a model for a sequence of morphological changes, named the PanIN system [6], in which the lower grade PanIN is thought to exchange to higher grade PanIN and finally to PDC [7-9]. And MGH with no atypia is almostly equal to PanIN-1A and MGH with atypia is almostly equal to PanIN-1B in this system [6-9].

The main purpose of the present study was to investigate the starting point of MGH as pre-cancerous lesion of PDC.

## Methods

Twenty-seven surgically resected pancreas tissue specimens, including 14 cases of PDC, which consisted of moderately differentiated tubular adenocarcinoma, 7 cases of the chronic pancreatitis and 6 cases of the normal pancreas, were assessed at the Department of Pathology, Juntendo University Shizuoka Hospital between 1998 and 2004. Informed consent for the medical examinations described below was obtained from all patients.

The specimens were fixed in 10% formalin solution for 1 – 5 days and prepared by cutting the lesions into 4 – 5 mm sections. Sections were embedded in paraffin, sectioned at a thickness of 4 μm and stained with hematoxylin and eosin (HE) and many immunohistochemical stainings described below. And after these stainings, some paraffin blocks were used for DNA extraction.

### 1. Immunohistochemical stainings were performed by the avidin-biotin-peroxidase complex method

anti-p53 oncoprotein (p53: DO7, monoclonal antibody, Novocastra Inc., UK), anti-MUC1 glycoprotein (MUC1: human CA 15-3, DF3, monoclonal antibody, DAKO, USA), anti-MUC6 glycoprotein (MUC6: CLH5, monoclonal antibody, Novocastra Inc., UK), anti-human gastric mucin-45M1 (45M1, monoclonal antibody, Novocastra Inc., UK) and anti-Ki-67 (MIB-1, monoclonal antibody, Coulter Japan Inc., Japan) (MUC1 and MUC6 at a dilution of 1:50, 45M1 at a dilution of 1:50, p53 at a dilution of 1:100 and Ki67 at a dilution of 1:100). Every staining was performed with 15-minute microwave treatment.

### 2. DNA extraction and analysis of K-ras mutation at codon 12

In 16 regions including the intercalated duct cells (IC) adjacent to the atypical MGH (panIN-1B), the existence or nonexistence of the K-ras codon 12 mutation was investigated, because the immunohistochemical expressions of the IC were very interesting, as described in the results, and K-ras codon 12 mutation is well known as one of the popular genetic abnormalities of PDC and MGH [10-13].

Paraffin blocks with the target foci were prepared for DNA extraction. The target foci were microdissected using a 20-gauge needle, comparing the slide with HE staining of same position. Extracted DNA was diluted with 5 ml of TaKaRa DEXPAT (for DNA Extraction from Paraffin-embedded Tissue, TaKaRa Biomedical Inc.).

Mutation of K-ras codon 12 was analized and compared in enriched polymerase chain reaction-enzyme-linked minisequence assay (PCR-ELMA) [14,15]. In PCR-ELMA, upstream for the first and second PCR were 5'-TAAACTTGTGGTAGTTGGAACT-3', downstream for the first PCR was 5'-GTTGGATCATATTCGTCCAC-3', and downstream for the second PCR was 5'-CAAATGATCTGAATTAGCTG-3'. The first PCR reaction was performed containing 1 μL of DNA lysate, 100 μM dNTP, 1.5 mM MgCl_2_, 1 μM each primers, 0.625 U Taq DNA polymerase and 1 × PCR buffer [containing 10 mM Tris-HCl (pH 8.3 at 25°C), 50 mM KCl and 0.001%(w/v) gelatin] in thermal cycler. And 10 μL of the denatured second PCR product was hybridized with probes for detecting the K-ras codon 12 wild-type (GGT) and six mutants (GAT, GCT, GTT, AGT, CGT and TGT) DNA were immobilized, at 55°C for 30 minutes, and 100 μL of biotinated A and 0.01 U of TdqDNA polypmerase were added and incubatuon was continued at 55°C for 30 minutes.

**Figure 1:**
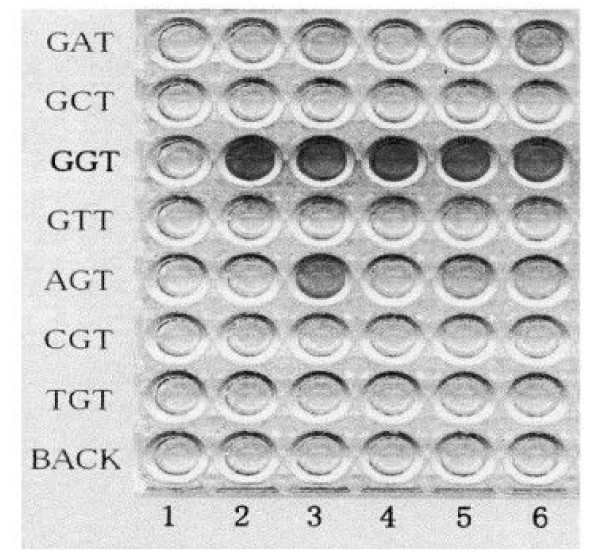
Enriched polymerase chain reaction – enzyme linked mini-sequence assay (PCR-ELMA), showing K-ras codon 12 mutation in mucous gland hyperplasia of the pancreas. AGT – type mutation was seen in lane 3.

In development, 100 μL of avidine-horseradish peroxidase conjugate was contained and the mixture was performed at room temperature for 30 minutes, and 100 μL of tetramethylbenzidine (TMB) substrate was containd and the plates were performed in the dark at room temperature for 20 minutes. And 100 μL of stop solution was contained and the light absorbance of each sample was measured by spectrophotometry (Multiskan Multisoft, Labsystems, Tokyo) with a 450 nm filter wavelength (Figure 1).

## Results

### 1. Immunohistochemical studies of pancreas tissue (Table 1)

**Table 1:** Positivity of Pancreatic Epithelium for Several Stainings

	MUC1	MUC6	45M1	Ki67	P53
Acinal cell (100 regions)	-	±	-	-	-
Intercalated duct (100 regions)	+	±	-	-	-
Duct (100 regions)	-	±	-	-	-
MGH with no atypia (50 regions)	-	+	+	±	-
Atypical MGH (20 regions)	+	+	+	±	-
Ductal carcinoma (15 lesions)	+	±	+	+	+

MGH: Mucous gland hyperplasia -: negative, +: positive, ±: positive or negative

**Figure 2:**
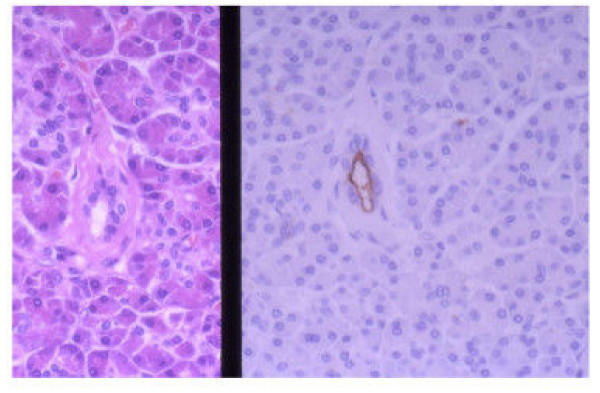
In normal pancreas, only the intercalated duct cells were positive for MUC1. (Left: HE, Right: MUC1, original magnification × 400)

In the normal pancreas (acinal cell: 100 regions, IC: 100 regions, duct: 100 regions), although no positive cell was seen in 45M1, p53, Ki67, the cytoplasm of IC were always positive for MUC1 (Figure 2) and sometimes positive for MUC6.

**Figure 3:**
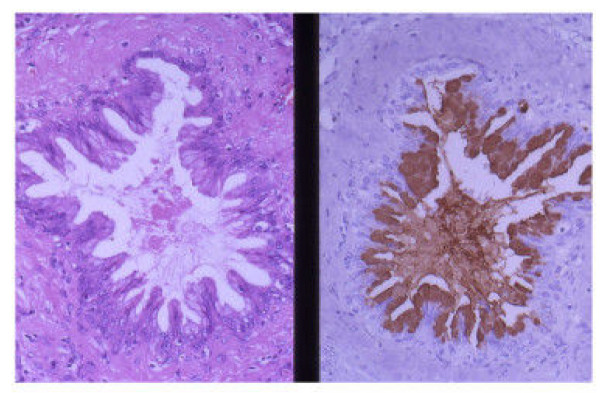
The epithelium of the mucous gland hyperplasia was positive for 45M1. (Left: HE, Right: 45M1, original magnification × 200)

**Figure 4:**
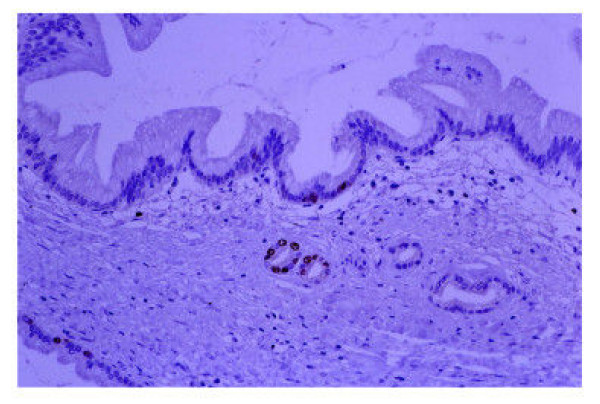
The intercalated duct cells adjacent to the atypical mucous gland hyperplasia were positive for Ki67. (Ki67, original magnification × 200)

In the pancreas with fibrosis or inflammation, the cytoplasm of the epitheli in MGH [16-18] with both no atypia (50 regions) and atypia (20 regions) was positive for 45M1 (Figure 3) and MUC6. And these atypical MGH (20 regions), which were PanIN-1B by PanIN system [1], were also positive for MUC1. No positive cell for p53 was seen in MGH with atypia or no atypia. Some IC adjacent to the atypical MGH (PanIN-1B) was positive for Ki67 (Figure 4).

The carcinoma cells in all cases (14 cases) of PIDC were diffusely positive for MUC1, 45M1, p53 and Ki67, and focally positive for MUC6. Namely, in all cases, the nuclei of many carcinoma cells were positive for p53 and Ki67. And in all cases, the expression of MUC1, MUC6 and 45M1 apomucins was found in the cell membranes of many carcinoma cells and the cytoplasms of some carcinoma cells.

### 2. K-ras mutation

In K-ras mutation, we examined the regions including IC adjacent to the atypical MGH, because the immuno-histochemical apomucins stainings of these regions were almostly equal to those of PDC described above. And K-ras mutation was comfirmed in 12 of 16 regions including IC adjacent to the atypical MGH (75%). All mutations were a single mutation, in 6 regions GTT was detected, in 4 regions GAT was detected and in 2 region AGT was detected.

## Discussion

Generally, the neoplasia or pre-neoplasia will have some characteristics of its original cell and may develop from the proliferative cell in the organ. Thus, it is important to find out the normal cells which have the characteristics of the neoplasia or pre-neoplasia for detecting the starting point of the neoplasia.

Therefore, in the present study, the mucin expressions which are known as one of the characteristics of PDC [19-24] and K-ras mutation which is very famous gene abnormality in PDC [10,11] were investigated the non-neoplastic tissue or the neoplasia.

Although the results in the current study may be little novel and supportive of the known facts, these findings should reveal that the present study was correct.

That is to say, in the current study, the intercalated duct cells (IC) were positive for MUC1, although the other normal epitheli of the pancreas were negative for it. In the other reports, the positivity for MUC1 in the normal pancreas lack consistency, positive [22] or negative [21,23]. The reason of this discrepancy is unkown, however, in this study, the normal pancreas tissues were obtained from surgical resection due to non-pancreatic diseases and these specimens were quickly fixed in formalin solution. Thus, these specimens were suitable for the detailed histological examination in the normal pancreas and the results in the current study should be reliable.

And the results in the present study indicated that the mucin phenotypes of the atypical MGH (PanIN-1B by PanIN system) had a diffusely positive reaction for both anti-MUC1 staining and anti-45M1 staining, and had a spasely positive reaction for anti-Ki-67 staining, and these findings resembled those of the pancreatic ductal carcinoma (PDC), except for the expression of p53 (MGH: negative, PDC: positive). The expressions of the apomucins, Ki67 and p53 in MGH and PDC in the present study matched mostly those of other reports [20,21,23-26].

It has been well known that the K-ras mutation may confirm in MGH and PDC [10-13] and the results in the current study also showed that the K-ras codon 12 mutations were confirmed in the regions including IC adjacent to the atypical MGH of human pancreas (GGT→GTT in 6 regions, GGT→GAT in 4 regions and GGT→AGT in 2 region). And some IC adjacent to the atypical MGH was positive for Ki67.

Namely, the IC adjacent to the atypical MGH (PanIN-1B), which is sometimes proliferative cell, had the mucin phenotypic expression and high frequency of K-ras mutation as well as PDC.

Thus, we think that some IC is the starting point of MGH and may be thought to be the starting point of PDC, considering the histogenesis of PDC (from lower grade PanIN to higher grade PanIN, to PDC).

Further molecular studies concerning the intercalated duct cell in various pancreatic disease should be warranted.

## Conclusion

Some intercalated duct cell may be the starting point of the pancreatic ductal carcinoma, because the exhibitions of mucin expressions, Ki67, p53 and K-ras mutation in some intercalated duct cell resembled those of mucous gland hyperplasia or pancreatic ductal carcinoma.

## Abbreviations

IC, intercalated duct cell ; PDC, pancreatic ductal carcinoma ; MGH, mucous gland hyperplasia

## References

[B1] Tsuchiya R, Tajima Y, Matsuzaki S (2001). Early pancreatic cancer. Pancreatology.

[B2] Egawa S, Takeda K, Fukuyama S (2004). Clinicopathological aspects of small pancreatic cancer. Pancreas.

[B3] Sommers SC, Murphy SA, Warren S (1954). Pancreatic duct hyperplasia and cancer. Gastroenterology.

[B4] Kozuka S, Sassa R, Taki T (1979). Relation of pancreatic duct hyperplasia to carcinoma. Cancer.

[B5] Chen J, Baithun SI, Ramsay MA (1985). Histogenesis of pancreatic carcinomas: a study based on 248 cases. J Pathol.

[B6] Hurban RH, Adsay NV, Albores-Saavedra J (2001). Pancreatic intraepithelial neoplasia: a new nomenclature and classification system for pancreatic duct lesions. Am J Surg Pathol.

[B7] Hruban RH, Wilentz RE, Maitra A (2004). Identification and analysis of precursors to invasive pancreatic cancer. Methods Mol Med.

[B8] Kloppel G, Luttges J (2004). The pathology of ductal-type pancreatic carcinomas and pancreatic intraepithelial neoplasia: insights for clinicians. Curr Gastroenterol Rep.

[B9] Hruban RH, Takaori K, Klimstra DS (2004). An illustrated consensus on the classification of pancreatic intraepithelial neoplasia and intraductal papillary mucinous neoplasms. Am J Surg Pathol.

[B10] Almoguera C, Shibata D, Forrester K (1988). Most human carcinomas of exocrine pancreas contain mutant c-K-ras genes. Cell.

[B11] Grunnewald K, Lyons J, Frohlich A (1989). High frequency of Ki-ras codon 12 mutations in pancreatic adenocarcinomas. Int J Cancer.

[B12] Tabata T, Fujimori T, Maeda S (1993). The role of ras mutation in pancreatic cancer, precancerous lesions, and chronic pancreatitis. Int J Pancreatol.

[B13] Yanagisawa A, Ohtake K, Ohashi K (1993). Frequent c-Ki-ras oncogene activation in mucous cell hyperplasias of pancreas suffering from chronic inflammation. Cancer Res.

[B14] Matsubayashi H, Watanabe H, Yamaguchi T (1999). Difference in mucus and K-ras mutation in relation to phenotypes of tumors of the papilla of Vater. Cancer.

[B15] Wada R, Yamaguchi T (2003). K-ras codon 12 mutations of the super-minute dysplasia in Barrett's esophagus by DNA extraction using a microdissection method. Dis Esophagus.

[B16] Walters MN (1965). Goblet-cell metaplasia in ductules and acini of the exocrine pancreas. J Pathol Bacteriol.

[B17] Pour PM, Sayed S, Sayed G (1982). Hyperplastic preneoplastic, and neoplastic lesions found in 83 human pancreas. Am J Clin Pathol.

[B18] Cubilla AL, Fitzgerald PJ (1984). Tumors of the exocrine pancreas. Atlas of Tumor Pathology, Fascile 19, 2nd Series.

[B19] Chambers JA, Hollingsworth MA, Trezise AE (1994). Development expression of mucin genes MUC1 and MUC2. J Cell Sci.

[B20] Terada T, Ohta T, Sasaki M (1996). Expression of MUC apomucins in normal pancreas and pancreatic tumours. J Pathol.

[B21] Masaki Y, Oka M, Ogura Y (1999). Sialylated MUC1 mucin expression in normal pancreas, benign pancreatic lesions, and pancreatic ductal adenocarcinoma. Hepatogastroenterology.

[B22] Balague C, Gambus G, Carrato C (1994). Altered expression of MUC2, MUC4, and MUC5 mucin genes in pancreas tissues and cancer cell lines. Gastroenterology.

[B23] Monges GM, Mathoulin-Portier MP, Acres RB (1999). Differential MUC1 expression in normal and neoplastic human pancreatic tissue. An immunohistochemical study of 60 samples. Am J Clin Pathol.

[B24] Yonezawa S, Horinouchi M, Osako M (1999). Gene expression of gastric type mucin (MUC5AC) in pancreatic tumors: its relationship with biological behavior of the tumor. Pathol Int.

[B25] Scarpa A, Capelli P, Mukai K (1993). Pancreatic adenocarcinomas frequently show p53 gene mutation. Am J Pathol.

[B26] Redston MS, Caldas C, Seymour AB (1994). p53 mutations in pancreatic carcinoma and evidence of common involvement of homocopolymer tract in DNA microdeletions. Cancer Res.

